# The impact of reproductive factors on DNA methylation-based telomere length in healthy breast tissue

**DOI:** 10.1038/s41523-022-00410-4

**Published:** 2022-04-13

**Authors:** Mary E. Sehl, Jill E. Henry, Anna Maria Storniolo, Steve Horvath, Patricia A. Ganz

**Affiliations:** 1 Medicine, Hematology-Oncology, David Geffen School of Medicine, University of California Los Angeles, Los Angeles, CA 90095 USA; 2 Computational Medicine, David Geffen School of Medicine, University of California Los Angeles, Los Angeles, CA 90095 USA; 3 Susan G. Komen Tissue Bank at the Indiana University Simon Cancer Center, Indianapolis, IN 46202 USA; 4 Biostatistics, School of Public Health, University of California Los Angeles, Los Angeles, CA 90095 USA; 5 Department of Human Genetics, David Geffen School of Medicine, Gonda Research Center, University of California Los Angeles, Los Angeles, CA 90095 USA; 6 Health Policy and Management, Fielding School of Public Health, University of California Los Angeles, Los Angeles, CA 90095 USA; 7 UCLA-Jonsson Comprehensive Cancer Center, Los Angeles, USA

**Keywords:** Breast cancer, Epigenetics

## Abstract

Estrogen promotes breast tissue proliferation and telomerase activation. We investigated the effects of reproductive history on cell cycling and telomere length using a DNA methylation-based estimate of telomere length (DNAmTL) in breast and blood from healthy women donors. We demonstrate that DNAmTL is shorter in breast than in blood, and that nulliparous women have longer age-adjusted DNAmTL in both breast and blood, potentially explaining their higher risk of breast cancer.

## Introduction

Estrogens and progesterone play a major role in promoting proliferation of breast tissue during normal breast development, during pregnancy and lactation, and during breast carcinogenesis^1,2^. Telomere attrition leads to a proliferative barrier, as telomeres shorten in normal cells with each cell division, ultimately halting the ability of the cell to further divide, and this process can protect against cancer^3,4^. However, this barrier can be overcome when telomerase is activated. Telomerase activity has been demonstrated in normal breast tissue and pre-invasive lesions^5^. Estrogen has been shown to activate telomerase in tumor tissue^6,7^, and to stimulate telomerase activity in peripheral blood^8^. However, the effects of cumulative estrogen exposure on telomerase activity and telomere length in normal breast tissue are not known.

Recently, a DNA methylation-based estimator of telomere length (DNAmTL) was developed^9^. This epigenetic biomarker effectively captures the replicative history of cells and is highly predictive of age-related pathologies. Based on DNA methylation levels at 140 CpGs, this measure was developed in leukocytes and adipose tissue and is applicable across the entire age spectrum and across tissues including liver, adipose, and monocytes. In this study, we examine the relationship between lifetime estrogen exposure and cell replication potential in breast tissue, using DNAmTL. We hypothesize that chronic cell cycling during breast development, pregnancy, and lactation leads to shortened telomeres, while increased estrogen signaling and telomerase activation may contribute to longer telomeres in breast in the absence of pregnancy and lactation. We compare DNAmTL in breast and blood, using paired samples from 40 women donors. Finally, we examine the effects of lifetime estrogen exposure, reproduction and lactation on breast DNAmTL using data from a larger study of 192 women with self-reported variables on menstrual status, reproductive and lactation history, and exposure to exogenous hormones.

## Results

### Comparing DNAmTL in breast and blood

**Fig. 1:**
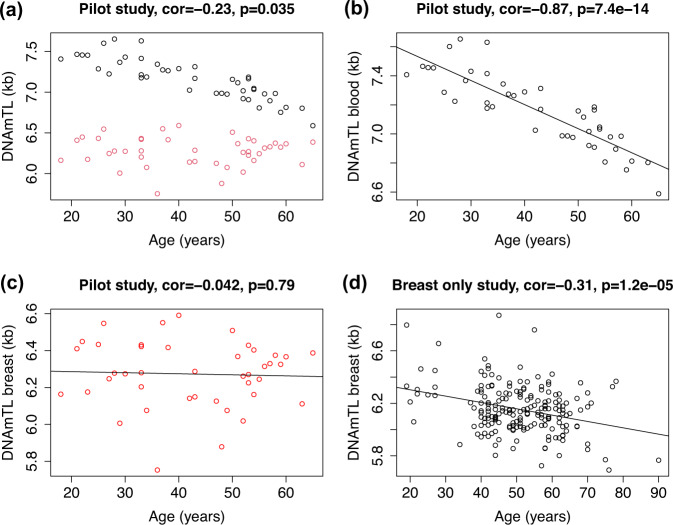
Comparison of DNA methylation-based estimates of telomere length (DNAmTL) in blood (black circles) and breast (red circles). Panel **a** reveals a scatter plot of chronologic age versus DNAmTL in blood and breast tissue from 40 women with paired samples. Regression lines demonstrate the inverse linear relationship between DNAmTL and chronologic age in blood (panel **b**) and breast (panel **c**) in these 40 women. Using data from a larger study of breast samples only (*N* = 192 women), a significant inverse relationship is demonstrated between DNAmTL and chronologic age in breast (Panel **d**).

Figure 1 demonstrates the inverse relationship between DNAmTL and chronological age in breast and blood. DNAmTL is strongly inversely correlated with chronologic age in peripheral blood (cor = −0.85, p < 0.0001). While we did not identify a correlation between breast DNAmTL in our smaller cohort, there was a significant inverse correlation between breast DNAmTL and chronologic age (cor = −0.31, *p* < 0.0001) in the larger sample (*N* = 192).

We examined tissue differences in DNAmTL in breast compared with blood (*N* = 40) (Supplementary Fig. 1). In all samples, we found that the cell replicative history was significantly advanced in breast compared with blood at earlier ages. This difference is more pronounced at earlier ages closer to puberty and development, suggesting that hormones may play a role in this difference.

### Factors associated with DNAmTL in blood

**Fig. 2:**
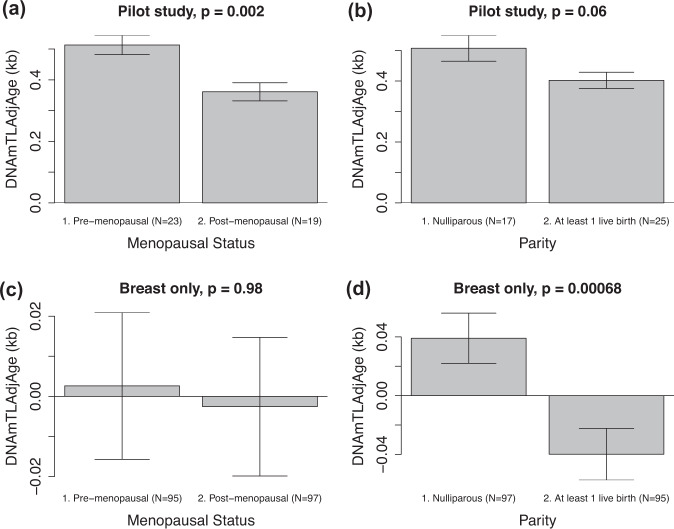
Age-adjusted DNAmTL by menopausal status and parity. Bar plots demonstrate the relationship between age-adjusted DNAmTL and menopausal status in blood (panel **a**) and breast (panel **c**), and the relationship between age-adjusted DNAmTL and parity in blood (panel **b**) and breast (panel **d**). These plots demonstrate the mean value (*y*-axis) and error bars indicate one standard error; with *p*-values from the results of a non-parametric group comparison test (Kruskal–Wallis).

Of the 40 women in the smaller cohort, 17 were nulliparous, and 23 were pre-menopausal. When we examine the age-adjusted DNAmTL measure (adjusted for age by taking the residuals of a linear regression of DNAmTL on chronologic age for both breast and blood samples at the first visit) in peripheral blood of pre-menopausal and post-menopausal women, non-parametric group comparisons demonstrate shorter age-adjusted DNAmTL in post- vs. pre-menopausal, women (see Fig. 2). At visit 1, the 17 women who were nulliparous had never been pregnant. Women with ≥1 live birth tended to have shorter age-adjusted DNAmTL than nulliparous women, though this finding did not meet statistical significance (*p* = 0.06 using the non-parametric Kruskal–Wallis test).

### Factors associated with DNAmTL in breast

Using the larger (*N* = 192) data set, we examined for reproductive and hormonal factors associated with DNAmTL. In this larger study, 94 women were nulliparous, and 97 were post-menopausal. Supplementary Table 1 and Fig. 2 reveal the significant relationships between age-adjusted breast DNAmTL and gravidity, parity, and breastfeeding. Nulliparity was significantly associated with longer estimated telomere length (*β* = 0.080, *p* = 0.0015). With each birth, DNAmTL decreased by −0.023 units, after adjusting for chronologic age (*p* = 0.013). Likewise, having never been pregnant was associated with longer estimated telomere length (*β* = 0.079, *p* = 0.0019). With each pregnancy, DNAmTL decreased by −0.018 units (*p* = 0.017). While having ever breastfed was significantly associated with shorter DNAmTL in models adjusted for chronologic age, the majority (71) of the 95 women who had given birth had also breastfed. In models adjusted for both chronologic age and having ≥1 live birth, having ever breastfed was no longer associated with DNAmTL (*β* = 0.021, *p* = 0.61). Menopausal status was not associated with age-adjusted DNAmTL. Women who used birth control pills tended to have longer DNAmTL after adjusting for chronologic age (*β* = 0.063, *p* = 0.082) though this finding did not reach significance. There was no association between hormone replacement therapy and DNAmTL. In order to examine whether DNAmTL is related to self-reported measures of cumulative estrogen exposure, we examined the relationship between DNAmTL and age at menarche and age at first full term birth. We did not find a significant association between earlier age at menarche and DNAmTL after adjusting for chronologic age. When we limited our sample to only those women (*N* = 94) who have had at least 1 live birth, we did not find an association between breast DNAmTL and age at first live birth (*β* = −0.00027 units per year of age, *p* = 0.84). These findings suggest that DNAmTL captures features of cell replicative history that are distinct from those captured by patient history and other biomarkers.

## Discussion

We find that DNAmTL is shorter in breast than peripheral blood, suggesting that cell replicative aging of breast is more advanced than that of peripheral blood, particularly in early adulthood. In breast and blood, DNAmTL is significantly shorter in women undergoing pregnancy, childbirth, and lactation. Proliferation and differentiation are part of normal breast development, and also occur during periods of pregnancy and lactation^1,2^. Our results suggest that these events in breast prior in the years leading up to and directly following menarche may account for the accelerated shortening of telomere length in breast compared with blood that we observe in early adulthood. Likewise, proliferation of breast tissues during pregnancy and lactation may explain the accelerated shortening of telomeres we observe with each pregnancy and live birth and with duration of breastfeeding. Nulliparous women have prolonged exposure to estrogen and higher risk of estrogen receptor positive breast cancer^10–14^. Our finding that telomere length is longer in nulliparous women raises the question of whether telomerase is activated in these women and whether this activation is mediated by prolonged estrogen signaling. Alternatively, it may reflect fewer cell divisions in nulliparous women.

Estrogen deficiency^15^ and shorter duration of reproductive years^16^ have been associated with shorter leukocyte telomere length (LTL) in peripheral blood. Our finding of shorter age-adjusted DNAmTL in the peripheral blood of post-menopausal compared with pre-menopausal women is consistent with these prior studies of direct measurement of LTL in lower estrogen states. In addition, previous studies have demonstrated that women with late maternal age have longer LTL^17^, and LTL has been shown to be 4.2% shorter in parous compared with nulliparous women^18^. Our findings of shorter DNAmTL in women with history of pregnancy and childbirth are consistent with these studies. However, we further show in this study that estimated telomere length also shortens in breast tissue with rising gravidity and parity.

Limitations of our study include the small sample size of women with peripheral blood measurements, and the use of a biomarker not developed in breast. We caution the reader that while DNAmTL has a relatively weak correlation with LTL, shorter DNAmTL is a much better predictor of time to death, and is more strongly correlated with age, BMI, tobacco smoking, and earlier age at menopause^9^.

Future work is needed to evaluate whether DNA methylation-based estimates of telomere length are associated with known breast cancer risk models. In our cross-sectional cohort (*N* = 192), we found no correlation between longer age-adjusted DNAmTL and higher Gail scores (*β* = 3.25, *p* = 0.21), or higher 10-year (*β* = 1.78, *p* = 0.11), and lifetime (*β* = 4.36, *p* = 0.15) Tyrer–Cuzick risk estimates. Larger studies are needed to examine this question, as methylation-based estimates of cell replication history may provide a unique risk assessment in an individual patient that may guide germline testing algorithms. Notably, there were six participants in our data set (four from the pilot paired breast/blood data and two from the larger data set) who were later found to carry germline mutations on whole genome sequencing—2 with BRCA1 mutations, 1 with an ATM mutation, and 3 with PALB2 mutations. Lymphocyte telomere length is prolonged in carriers of BRCA1 and BRCA2 mutations^19^, and longer telomere length in blood is predictive of breast cancer risk in BRCA1 and BRCA2 mutation carriers^20^. However, no studies have previously examined telomere length in high risk patients. Future studies are needed to examine when at women at high risk of familial breast cancer have significant alterations in methylation-based estimates of telomere length in breast tissue.

The relationship between breast tissue aging, estrogen signaling, cellular proliferation, carcinogenesis, and replicative senescence is complex. While cellular senescence is an effective mechanism for tumor suppression, the senescence-associated secretory phenotype is associated with factors that promote chronic inflammation and hyperplastic pathologies^21^. Future studies are needed to determine whether telomerase activation is present in pre-malignant lesions and breast tissues of women at high risk for breast cancer. Recent studies have shown that families with loss of the telomere suppressor pathway caused by inherited mutations in TINF2, a gene that controls telomere length, have an increased risk of breast and other cancers^3,4^. Further work is needed to examine genetic and environmental factors that modulate telomerase expression in breast tissue and to develop targeted interventions for prevention.

## Methods

### Study specimens

The Susan G. Komen Tissue Bank (KTB) at the Indiana University Simon Cancer Center is a unique resource developed with the goal of understanding normal breast tissue biology to better understand and prevent breast carcinogenesis^22^. Partipants in the KTB have provided informed consent. This study was approved by the UCLA Institutional Review Board. Data for this study were previously collected as part of two studies designed to examine epigenetic age in healthy breast tissue^23,24^. In the pilot longitudinal study, DNA methylation age was compared in breast and blood using paired samples from the same 40 individuals at two ore more time points^23^. We draw data from the initial visit of this study. The second, larger cross-sectional study was designed to examine associations between breast DNA methylation age and hormonal factors, using breast tissue samples from 192 women^24^. In both studies, we selected samples to have balanced representation from pre- and post-menopausal women, and from women with and without ≥1 live birth. Details on tissue processing, DNA extraction and methylation experiments are provided in the Supplementary Methods section of the Supplemental Material.

### Statistical analyses

Because of the small sample size (*N* = 40) of the longitudinal cohort, nonparametric testing was used to test for differences in age-adjusted DNAmTL (formed using the residuals from a linear regression of DNAmTL on chronologic age) in peripheral blood between pre- vs. post-menopausal women, and between those with and without a history of pregnancy and childbirth. In the larger breast-only sample (*N* = 192), multivariate linear regression models were used to examine for associations between breast DNAmTL and covariates of interest including ethnicity, body mass index (BMI), age at menarche, menopausal status, age at menopause, gravidity, parity, age at first live birth, duration of breastfeeding, use of oral contraceptives, and hormone replacement. All models were adjusted for age at tissue donation.

### Reporting summary

Further information on research design is available in the Nature Research Reporting Summary linked to this article.

## Supplementary information

Supplementary Material

Reporting Summary Checklist

## Data Availability

The data generated and analyzed during this study are available through the Susan G. Komen Virtual Tissue Bank (https://virtualtissuebank.iu.edu/), an online public repository resource freely available comprising all raw data generated by investigators working with samples from KTB participants.

## Code availability

Software used in the data analyses of this manuscript will be made freely available through GitHub for readers to access and download.
