# Supplementary material for: The impact of reproductive factors on DNA methylation-based telomere length in healthy breast tissue
Source: NPJ Breast Cancer. 2022 Apr 13;8:48. doi: 10.1038/s41523-022-00410-4 (PMC9007943; doi:10.1038/s41523-022-00410-4)
Supplement: Supplementary file 1 — Supplementary Material [file 41523_2022_410_MOESM1_ESM.pdf]

Supplementary Figures

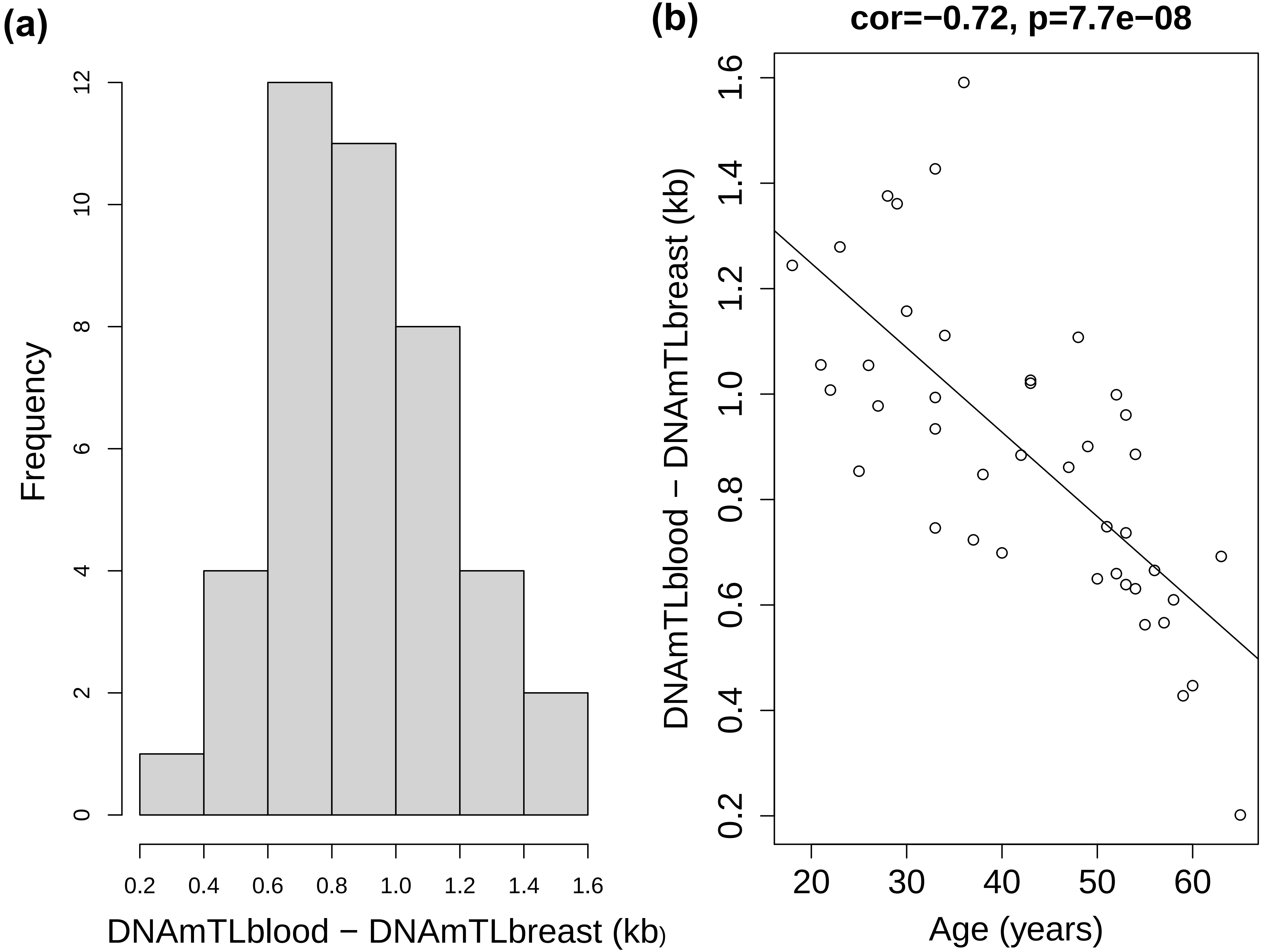

Supplementary Figure 1. Difference in estimated telomere length (DNAmTL) in blood and breast tissues. DNAmTL was shorter in breast than in blood, in all paired samples from 40 healthy women donors (Panel (a)). The difference between DNAmTL in blood and breast was significantly higher at earlier ages closer to menarche, and this difference declined with advancing age (Panel (b)).

Supplementary Tables

Supplementary Table 1. Hormonal and reproductive factors associated with DNAmTL*

	**β**	**p**
**Ethnicity**		
**Hispanic**	-0.035	0.56
**Tobacco use**		
**Ever**	0.039	0.17
**Years smoked**	-0.0025	0.21
**Body mass index**	-0.00019	0.92
**Age at menarche**	-0.0013	0.87
**Premenopausal**	0.010	0.77
**Age at menopause**	0.0022	0.39
**Gravidity**		
No history of pregnancy	**0.079**	**0.0019**
**Parity**		
Nulliparous	**0.080**	**0.0015**
**Age at first live birth**	-0.00027	0.94
**Breastfeeding**		
**Ever**	**-0.055**	**0.032**
**Total months**	-0.0015	0.13
**Birth control**	0.064	0.080
**Hormone replacement**	0.013	0.70

*All models adjusted for chronologic age.

Supplementary Methods

Tissue processing, DNA extraction, and DNA methylation studies

Methods describing breast tissue and blood sample acquisition and processing, DNA isolation, and bisulfite sequencing experiments are detailed in previous reports (11,12). Briefly, breast and peripheral blood tissues were collected from donors to the Komen Tissue Bank. In the first study cohort, paired breast and blood were collected (N=40 pairs at two or more time points), and in the second cohort only breast tissues were collected (N=200). At the Komen Tissue Bank, for the first study, blood was drawn into EDTA 9ml blood collection tubes. Plasma was removed by centrifugation, and red cells and buffy coat were stored at -80 °C . DNA extraction was performed at Indiana CTSI Specimen Storage Facility lab using an AutogenFlex Star (SN 401033) instrument and the Flexigene AGF3000 blood kit for DNA extractions from whole fresh and frozen blood. For both studies, six core samples were taken from the upper outer quadrant of the breast of the donor under local anesthesia. While one of the core biopsies was placed into an embedding cassette within five minutes of procurement, placed in 10% buffered formalin, embedded in paraffin, and stored at room temperature, the remaining five core biopsies were flash frozen in liquid nitrogen, placed in labeled cryovials, stored at -166.2 °C. The cryovials were sent to UCLA UNGC core where the AllPrep DNA/RNA/miRNA Universal Kit was used to extract DNA from frozen tissue samples. Extracted DNA was then used for bisulfite sequencing experiments. Bisulfite sequencing experiments for blood and breast were performed at the UCLA Neurogenetics Core Laboratory. Bisulfite conversion was performed using the Zymo EZ DNA

Methylation Kit. 500 ng DNA was bisulfate-converted using the EZ-methylation kit (Zymo Research) in both studies. In the first study, bisulfite-converted DNA was hybridized to the Infinium HumanMethylation450 BeadChip (Illumina, San Diego, CA), and in the second larger study, bisulfite-converted DNA was hybridized to Infinium Human Methylation EPIC (850K) array (Illumina, San Diego, CA). Fluorescence data from the hybridized chip were scanned on an iScan (Illumina) and analyzed. DNA methylation levels (beta-values) were determined by calculating the ratio of intensities between methylated (signal A) and un-mehtylated (signal B) sites. We used the "noob" normalization method implemented in the minfi R package. Specifically, the beta value was calculated from the intensity of the methylated (M corresponding to signal A) and un-methylated (U corresponding to signal B) sites, as the ratio of fluorescent signals beta = Max(M,0)/[Max(M,0) + Max(U,0) + 100]. Thus, beta values range form 0 (completely un-methylated) to 1 (completely methylated).
