# Supplementary material for: The impact of reproductive factors on DNA methylation-based telomere length in healthy breast tissue
Source: NPJ Breast Cancer. 2022 Apr 13;8:48. doi: 10.1038/s41523-022-00410-4 (PMC9007943; doi:10.1038/s41523-022-00410-4)
Supplement: Supplementary file 2 — Reporting Summary Checklist [file 41523_2022_410_MOESM2_ESM.pdf]

nature portfolio

Corresponding author(s): Mary E. Sehl, MD, PhD
Last updated by author(s): Aug 25, 2021

Reporting Summary

Nature Portfolio wishes to improve the reproducibility of the work that we publish. This form provides structure for consistency and transparency in reporting. For further information on Nature Portfolio policies, see our Editorial Policies and the Editorial Policy Checklist.

Statistics

For all statistical analyses, confirm that the following items are present in the figure legend, table legend, main text, or Methods section.

n/a	Confirmed	
☐	☒	The exact sample size (*n*) for each experimental group/condition, given as a discrete number and unit of measurement
☐	☒	A statement on whether measurements were taken from distinct samples or whether the same sample was measured repeatedly
☐	☒	The statistical test(s) used AND whether they are one- or two-sided<br>*Only common tests should be described solely by name; describe more complex techniques in the Methods section.*
☐	☒	A description of all covariates tested
☐	☒	A description of any assumptions or corrections, such as tests of normality and adjustment for multiple comparisons
☐	☒	A full description of the statistical parameters including central tendency (e.g. means) or other basic estimates (e.g. regression coefficient) AND variation (e.g. standard deviation) or associated estimates of uncertainty (e.g. confidence intervals)
☐	☒	For null hypothesis testing, the test statistic (e.g. *F*, *t*, *r*) with confidence intervals, effect sizes, degrees of freedom and *P* value noted<br>*Give P values as exact values whenever suitable.*
☒	☐	For Bayesian analysis, information on the choice of priors and Markov chain Monte Carlo settings
☒	☐	For hierarchical and complex designs, identification of the appropriate level for tests and full reporting of outcomes
☐	☒	Estimates of effect sizes (e.g. Cohen's *d*, Pearson's *r*), indicating how they were calculated

Our web collection on statistics for biologists contains articles on many of the points above.

Software and code

Policy information about availability of computer code

Data collection	Data were stored and managed using R statical software, version 4.0.2.
Data analysis	Data were analyzed using the DNA Methylation Age Calculator (dnamage.genetics.ucla.edu), and using standard regression packages in R statistical software, version 4.0.2 (www.rproject.org).

For manuscripts utilizing custom algorithms or software that are central to the research but not yet described in published literature, software must be made available to editors and reviewers. We strongly encourage code deposition in a community repository (e.g. GitHub). See the Nature Portfolio guidelines for submitting code & software for further information.

Data

Policy information about availability of data

All manuscripts must include a data availability statement. This statement should provide the following information, where applicable:
- Accession codes, unique identifiers, or web links for publicly available datasets
- A description of any restrictions on data availability
- For clinical datasets or third party data, please ensure that the statement adheres to our policy

The methylation data generated and analyzed during this study will be deposited in NCBI's Gene Expression Omnibus and will be made accessible through the GEO Series.

Field-specific reporting

Please select the one below that is the best fit for your research. If you are not sure, read the appropriate sections before making your selection.

☒ Life sciences ☐ Behavioural & social sciences ☐ Ecological, evolutionary & environmental sciences

For a reference copy of the document with all sections, see nature.com/documents/nr-reporting-summary-flat.pdf

Life sciences study design

All studies must disclose on these points even when the disclosure is negative.

Sample size	Sample size of 40 women was selected based on its ability to detect methylation pattern differences of 2-3 SD observed between breast tissue and blood tissue, and for the larger study a sample size of 192 was selected based on its ability to detect associations between methylation age and covariates related to lifetime estrogen exposure.
Data exclusions	We excluded data from 8 women who did not have adequate quantities of DNA isolated from their frozen breast tissue.
Replication	Data analyzed in our study included a longitudinal cohort (N=40) of women who donated both breast tissue and peripheral blood at two visits at least 1 year apart, and a larger cross-sectional study (N=192) of women who donated breast tissue only.
Randomization	This was an observational study on stored specimens and did not utilize randomization.
Blinding	This was an observational study on stored specimens and did not utilize blinding.

Reporting for specific materials, systems and methods

We require information from authors about some types of materials, experimental systems and methods used in many studies. Here, indicate whether each material, system or method listed is relevant to your study. If you are not sure if a list item applies to your research, read the appropriate section before selecting a response.

Materials & experimental systems

n/a	Involved in the study	
☒	☐	Antibodies
☒	☐	Eukaryotic cell lines
☒	☐	Palaeontology and archaeology
☒	☐	Animals and other organisms
☐	☒	Human research participants
☒	☐	Clinical data
☒	☐	Dual use research of concern

Methods

n/a	Involved in the study	
☒	☐	ChIP-seq
☒	☐	Flow cytometry
☒	☐	MRI-based neuroimaging

Human research participants

Policy information about studies involving human research participants

Population characteristics	We requested samples from pilot cohort (N=40) and larger cohort (N=200) healthy women aged 19-90 years, with selection of women from four groups: 1) pre-menopausal and nulliparous, 2) pre-menopausal and with at least 1 live birth, 3) post-menopausal and nulliparous, and 4) post-menopausal and with at least 1 live birth.
Recruitment	Study specimens and annotated data with information about the donor's age, ethnicity, height, weight, family history, medical history, reproductive history, and medication use were taken from the Susan G. Komen Tissue Bank at the Indiana University Simon Cancer Center, a unique repository and resource to breast cancer researchers.
Ethics oversight	This study was approved by the UCLA IRB (IRB # 16-000853 and IRB#14-001729).

Note that full information on the approval of the study protocol must also be provided in the manuscript.
